# Association of overexpressed carboxyl-terminal amyloid precursor protein in brains with altered glucose metabolism and liver toxicity

**DOI:** 10.1080/19768354.2023.2197761

**Published:** 2023-04-04

**Authors:** Sungguan Hong, Seungwoo Hong, Sung Hoon Lee

**Affiliations:** a Department of Chemistry, Chung-Ang University, Seoul, Republic of Korea; b College of Pharmacy, Chung-Ang University, Seoul, Republic of Korea

**Keywords:** *Carboxyl-terminal of amyloid precursor protein*, *liver*, *insulin/glucose tolerance*, *glucose and lipid metabolism-related genes*, *in vivo*

## Abstract

Alzheimer’s disease (AD) is the most prevalent neurodegenerative disease. The deposition of amyloid plaques mainly composed of amyloid beta (Aβ) is observed in brain regions in AD patients. AD presents with similar pathophysiology to that of metabolic syndrome, including glucose and insulin resistance. In addition, epidemiological studies indicate diabetes, impaired glucose metabolism, and obesity increase the prevalence of AD. The liver is considered a key organ in the reciprocal relationship between AD and metabolic syndrome and is the major organ for the clearance of Aβ in the periphery. Furthermore, liver dysfunction aggravates Aβ-induced pathophysiology. Aβ is produced in the brain and peripheral tissues and penetrates the blood–brain barrier. However, *in vivo* evidence showing the effect of Aβ on the crosstalk between the brain and liver has not been reported yet. In the present study, we investigated the toxicity of brain-derived Aβ on glucose metabolism and the liver using transgenic mice overexpressing the carboxyl-terminal of amyloid precursor protein in the brain. The transgenic mice were overweight, which was associated with impaired glucose metabolism and insulin resistance, but not due to increased food intake. In addition, transgenic mice had enlarged livers and reduced gene expressions associated with glucose and lipid metabolism. Thus, overexpressed amyloid precursor protein in the brain may promote being overweight and glucose resistance, possibly through liver toxicity.

## 1. Introduction

Alzheimer’s disease (AD) is a progressive neurodegenerative disorder leading to the loss of cognitive function. The deposition of amyloid beta (Aβ), a 39–43 amino acid long peptide derived from the cleavage of amyloid precursor protein (APP) (Vassar et al. 1999), is a hallmark of AD. APP has a single transmembrane domain with a large N-terminal extracellular domain and a short cytoplasmic domain. APP is cleaved by α-secretase to release sAPPα, and further cleavage of membrane-anchored C-terminal fragments (CTFs) by γ-secretase generates a soluble N-terminal fragment and membrane-bound C-terminal fragment (Tyan et al. 2012). However, the cleavage of APP by β-secretase releases sAPPβ and the further cleavage of CTFs by γ-secretase produces Aβ42. The overproduction and abnormal accumulation of Aβ as insoluble oligomers are relevant to AD (Hardy and Selkoe 2002).

The carboxyl-terminal 105 amino acid fragment of APP (APP-C100/C104), composed of an Aβ42 peptide and 58–62 adjacent amino acids, seems to contribute to the neuropathology of AD. The carboxyl-terminal 105 amino acid fragment of APP (C105) induces neurotoxicity in *Xenopus*, PC12 cells, and cortical neurons (Fraser et al. 1996, Kim and Suh 1996) and changes glutamatergic synaptic transmission in the cerebellar cortex (Hartell and Suh 2000). Injection of recombinant C105 caused memory impairment in mice and decreased ACh levels in the cortex and hippocampus (Choi et al. 2001). Furthermore, transgenic animals that overexpressed C105 in specifically in the brain by a neuron-specific enolase promoter, exhibited memory impairment and overexpression of Aβ42 in the brain (Lim et al. 2005, Lim et al. 2013). These studies indicate C105 may responsible for Aβ42-induced neurotoxicity and memory impairment during AD development.

Impaired glucose or lipid metabolism is a well-known risk factor for AD (Leibson et al. 1997, Ott et al. 1999). AD is referred to as type 3 diabetes (de la Monte 2019), and epidemiological studies have shown that diabetes patients exhibited lower cognitive function and a 2-3-fold increased risk for AD (Fontbonne et al. 2001, Biessels et al. 2006). ^18^F-deoxyglucose positron emission tomography studies have implicated the dysregulation of brain glucose uptake is associated with AD pathology (Mosconi 2005, Hunt et al. 2007), and impaired glucose metabolism and glycolytic flux in brains were also related to Aβ deposition and severity of AD (An et al. 2018). In addition, the abnormal dysregulation of brain glucose has been suggested to be an early marker of AD (Reiman et al. 2004, Herholz 2010), and longitudinal fasting plasma glucose was a primary observation of glucose dysregulation before the onset of clinical symptoms (An et al. 2018). Given that abnormal plasma glucose concentrations are associated with higher brain glucose concentrations in AD, abnormal plasma glucose concentrations may reflect the early stages of AD pathogenesis.

Lipid metabolism is also linked to AD because APP processing and Aβ production are involved in cholesterol metabolism (Wahrle et al. 2002, Grziwa et al. 2003). Elevated cholesterol is associated with AD development (Shepardson et al. 2011), and cholesterol levels correlated with Aβ production and burden (Refolo et al. 2001, Shie et al. 2002). Furthermore, lipid metabolism affected Aβ production and Aβ affected cholesterol metabolism or membrane fluidity (Grimm et al. 2005, Grimm et al. 2006).

It was suggested that high glucose or insulin resistance promoted Aβ production (Ho et al. 2004, Nagai et al. 2016), and that glucose facilitated the oligomerization of Aβ42 (Kedia et al. 2017). Aβ is known to inhibit the insulin pathway by reducing insulin binding and inducing insulin resistance by suppressing insulin receptors (Xie et al. 2002). In addition, Aβ impaired glucose uptake by the lipid peroxidation of transport protein 3 (GLUT3) in hippocampal and cortical neurons (Mark et al. 1997). These results indicate that Aβ and glucose or lipid metabolism have a reciprocal relationship. However, there is currently a lack of evidence from *in vivo* studies to support the claim that Aβ disrupts glucose or lipid metabolism. In the present study, we investigated Aβ-induced metabolism impairment using AD NSE/hAPP-C105 Tg mice, which exhibit the AD phenotype, and the selective expression of fragments of Aβ in brains to facilitate our understanding of the causal role of Aβ in abnormal glucose and lipid metabolism.

## 2. Materials and methods

### 2.1. Animal care

The animal care and experiments were performed in accordance with the guidelines issued by the Institutional Animal Care and Use Committee of Chung-Ang University. C57BL/6-Tg (NSE-hAPP-C105)/Korl (C105) mice were obtained from the National Institute of Food and Drug Safety Evaluation (NIFDS, Cheongju, Korea). The same number of mice were placed in one cage (3-4 mice/cage) with free access to water and standard rodent chow (PMI Nutrition, St. Louis, US) under a 12 h light/dark cycle. The same number of male and female mice was included per group for measuring mouse weight, food consumption, and tissue analysis.

### 2.2. Weight and food intake measurement

Mouse weight was measured every week. To measure food consumption, mice were individually housed in a standard cage, fasted for 6 h, and then food (5 g of chow) was supplied at 09:00 pm At 09:00 am, the remaining food was measured, and food consumption was calculated by subtracting the amount of remaining food from 5 g.

### 2.3. Glucose and insulin tolerance tests

Glucose or insulin tolerance tests were performed in accordance to a previous report with slight modification (Park et al. 2022). All animals were blinded prior to the glucose or insulin resistance tests. A glucose tolerance test (GTT) and insulin tolerance test (ITT) were performed after fasting for 12 h. Glucose was intraperitoneally (i.p.) injected at 1.5 g/kg of body weight for GTT, and insulin (Humulin R) was i.p. injected at 0.75 U/kg of body weight for ITT. Blood samples were collected from the tail vein at 0, 15, 30, 60, and 120 min after glucose or insulin injection, and blood glucose was determined by a glucometer (CareSens Pro, Seoul, Korea). Blood glucose levels were plotted against time.

### 2.4. Tissue preparation and qPCR

Total RNA was extracted from the liver tissues using TRIzol reagent. First-strand cDNA was synthesized by MultiScribe reverse transcriptase using random primers. Quantitative polymerase chain reaction (qPCR) was performed using Power SYBR Green Master Mix. Primer sequences used for the qPCR are listed in Supplementary Table 1. Glyceraldehyde 3-phosphate dehydrogenase (GAPDH) was used as the internal control for normalization. The relative quantitation of mRNA was determined based on the geometric mean of all the relative quantities of two internal control genes, with cycle threshold (Ct) values obtained using the QuantStudio1 Real-Time PCR System (Thermo Fisher Scientific, MA, USA).

### 2.5. Western blotting

Protein was extracted from liver tissue using RIPA buffer (Biosesang, Kyunggido, Republic of Korea) with phosphatase inhibitor cocktail (Roche, Basel, Switzerland). Protein concentrations were determined with bovine serum albumin (BSA) method. A total 20–40 μg of denatured protein was loaded and separated using 10% SDS-polyacrylamide gel electrophoresis. The proteins were transferred onto nitrocellulose membranes (GE Healthcare, Little Chalfont, Buckinghamshire, UK). The membranes were blocked by incubation of 5% skim milk in Tris-buffered saline (TBS) buffer containing 1% Tween 20 (TBS-T) at 20–25°C for 1 hr and then incubated with TBS-T containing primary antibody at 4°C overnight. The membranes were washed three times with TBS-T and incubated with secondary antibody at 20–25°C for 1 hr. The bands were developed with enhanced chemiluminescence (ECL) solution (WEST-ZOL Plus, iNtRON Biotechnology, Gyeonggi-do, Korea), and the bands were detected with a chemiluminescence system (Vilber, Marne-la-Vallée, France). Primary antibodies against peroxisome proliferator-activated receptor delta (PPARδ, 1:10,000; cat. no. #74076; Cell Signaling Technologies, Danvers, MA, USA), Akt (1:2,000; cat. no. #9272S; Cell Signaling Technologies), and GAPDH (1:10,000; cat. no. sc-25778, Santa Cruz Biotechnology, Dallas, TX, USA) were obtained from the indicated sources.

### 2.6. Statistical analysis

Data are presented as the mean value ± standard error of the mean (SEM). Statistical analyses were performed by Student’s *t*-tests and blood glucose levels were analyzed by one-way ANOVA followed by Tukey’s test. All analyses were performed using GraphPad Prism 5.01 (GraphPad software, La Jolla, CA, USA). *p* < 0.05 was considered statistically significant.

## 3. Results

### 3.1. Increased weight of C105 mice

**Figure 1.**
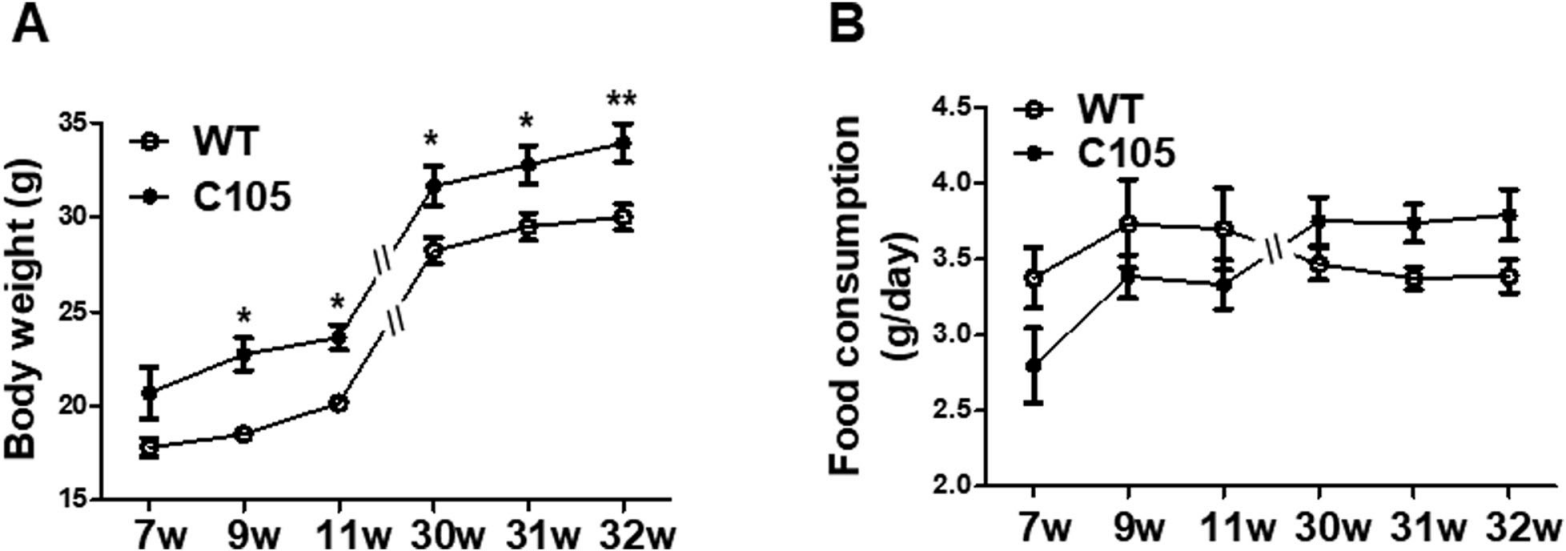
Body weight and food consumption of C105 mice. (A) Body weight of WT (*n* = 6–8) and C105 (*n* = 6–12) mice. (B) Food consumption per day of WT (*n* = 6–8) and C105 (*n* = 6–12) mice. Data shown are the mean ± SEM. *p < 0.05 and **p < 0.01 compared with WT mice.

To investigate whether C105 mice had metabolic dysfunction, we measured their weight weekly to compare body weight differences between the C105 and wild-type (WT) mice. To account for the fact that male mice typically have a higher weight than females, we include an equal number of males and females in each group when measuring animal weights. C105 mice exhibited a significantly higher weight from 9 weeks (7w: 20.68 ± 1.36, 9w: 22.74 ± 0.87, 11w: 23.65 ± 0.65, 30w: 31.64 ± 1.07, 31w: 32.76 ± 1.02, 32w: 33.92 ± 1.03) compared to the WT mice (7w: 17.81 ± 0.50, 9w: 18.50 ± 0.37, 11w: 20.16 ± 0.26, 30w: 28.21 ± 0.70, 31w: 29.48 ± 0.73, 32w: 30.00 ± 0.69) (Figure 1A). Next, we assessed whether increased food consumption induced weight gain in C105 mice. A significant difference in body weight was observed between C105 and WT for a period of 9 weeks, whereas the food uptake per day was similar in the WT (7w: 3.37 ± 0.19, 9w: 3.73 ± 0.29, 11w: 3.70 ± 0.27, 30w: 3.46 ± 0.10, 31w: 3.37 ± 0.07, 32w: 3.38 ± 0.10) and C105 groups (7w: 2.80 ± 0.24, 9w: 3.38 ± 0.14, 11w: 3.33 ± 0.16, 30w: 3.75 ± 0.15, 31w: 3.73 ± 0.12; 32w: 3.79 ± 0.16) (Figure 1B). These results suggest the increased weight of C105 mice may be related to the dysregulation of metabolism and not by food consumption.

### 3.2. Impaired glucose homeostasis in C105 mice

**Figure 2.**
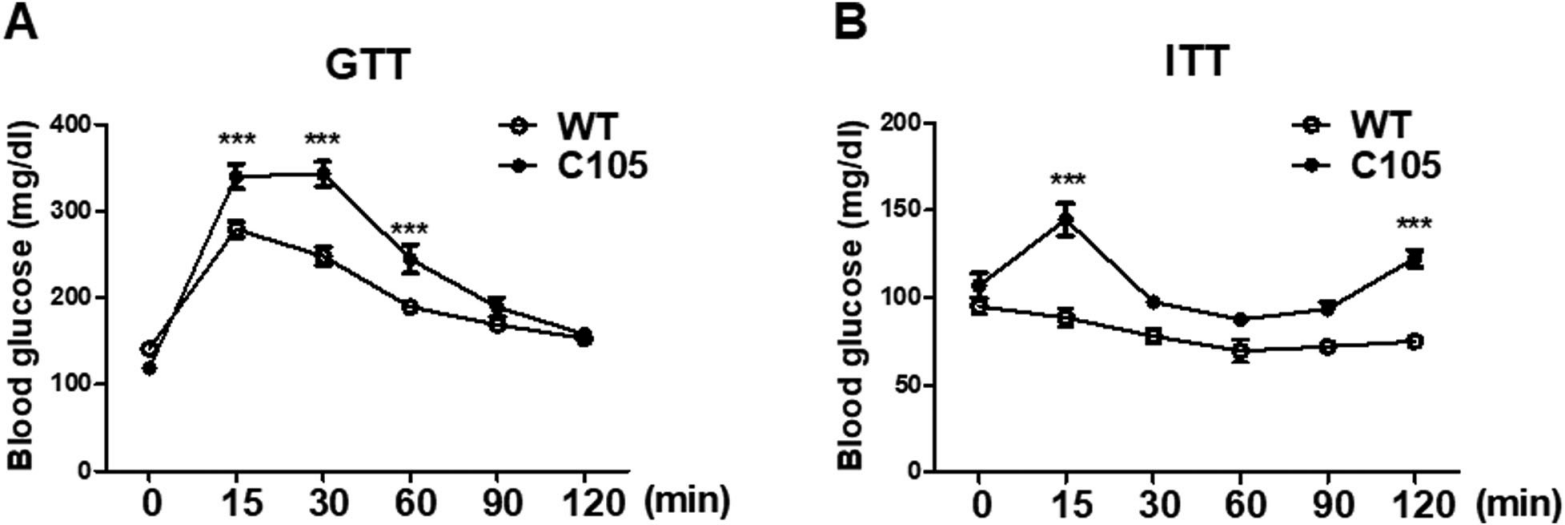
Glucose and insulin tolerance in C105 mice. (A) Glucose tolerance test in WT (*n* = 8) and C105 (*n* = 10) mice. Blood glucose measurements after glucose injection in WT and C105 mice. (B) Blood glucose measurements after insulin injection in WT (*n* = 8) and C105 (*n* = 10) mice. Data shown are the mean ± SEM. ***p < 0.001 compared with WT mice.

To investigate whether glucose metabolism was impaired in C105 mice, an *in vivo* GTT was performed by i.p. glucose injection after 12 h fasting. WT and C105 mice (9 weeks) were injected with glucose and plasma glucose concentrations were determined at various times. Blood glucose concentrations of WT were transiently increased by glucose injection, and subsequently decreased to baseline at 2 h (0 min: 141.23 ± 7.19, 15 min: 278.71 ± 9.11, 30 min: 247.66 ± 48.33, 60 min: 189.28 ± 6.90, 90 min: 168.76 ± 6.66, 120 min: 153.19 ± 6.67). C105 mice showed significantly impaired glucose tolerance (0 min: 118.87 ± 6.42, 15 min: 339.87 ± 14.44, 30 min: 342.68 ± 14.18, 60 min: 244.43 ± 16.52, 90 min: 189.06 ± 10.96, 120 min: 157.68 ± 6.50) (Figure 2A). To further investigate the insulin response of C105 mice, the kinetics of blood glucose were measured by glucose levels in insulin injected mice. C105 mice were significantly insensitive to insulin (Figure 2B; WT 0 min: 95.00 ± 4.00, 15 min: 88.50 ± 5.50, 30 min: 78.00 ± 4.00, 60 min: 69.50 ± 6.50, 90 min: 72.00 ± 3.00, 120 min: 75.00 ± 0.01; C105 0 min: 107.00 ± 7.00, 15 min: 144.67 ± 9.27, 30 min: 97.66 ± 1.76, 60 min: 87.67 ± 1.67, 90 min: 93.67 ± 3.92, 120 min: 122.67 ± 4.80) indicating C105 mice exhibited impaired glucose metabolism.

### 3.3. Increased liver weight in C105 mice

**Figure 3.**
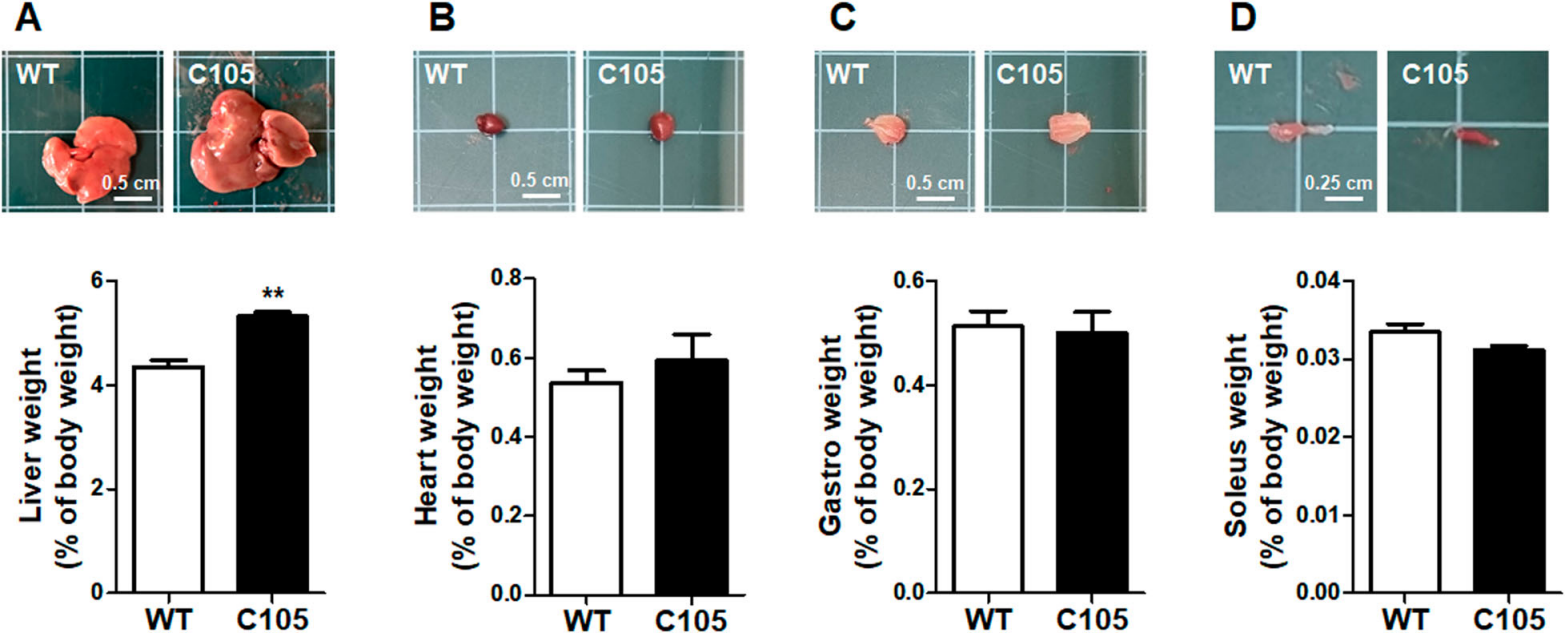
Organ weight of C105 mice. Liver, heart, gastro, and soleus weight of WT (*n* = 9) and C105 (*n* = 5) mice. **p < 0.01 compared with WT mice.

Next, we investigated the weight of organs of WT and C105 mice (9 weeks) involved in the regulation of glucose metabolism. Liver weight (WT: 4.34 ± 0.13; C105: 5.31 ± 0.08) and size were significantly increased in C105 mice (Figure 3), and the heart (WT: 0.53 ± 0.03; C105: 0.59 ± 0.06), gastrocnemius (WT: 0.51 ± 0.03; C105: 0.50 ± 0.04), and soleus (WT: 0.03 ± 0.001; C105: 0.03 ± 0.0005) were similar in WT and C105 mice.

### 3.4. Reduced glucose and lipid metabolism-related gene expressions in C105 mouse livers

**Figure 4.**
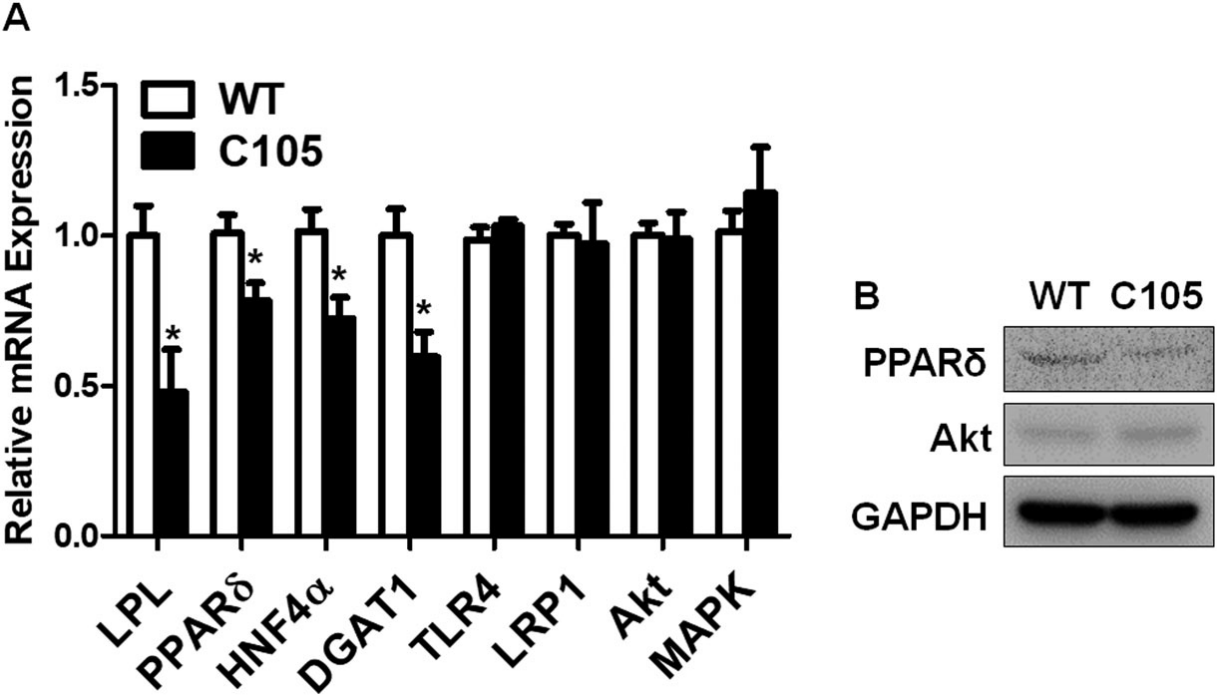
qPCR of genes and western blots of proteins. (A) Glucose and lipid regulating gene expressions were decreased in C105 mouse livers (*n* = 4–6 per group). *p < 0.05 compared with WT mice. (B) PPARδ and Akt protein expressions in mouse liver were analyzed with western blotting. GAPDH was used as a loading control.

Hepatic glucose metabolism is highly associated with lipid metabolism (Jones 2016), and insulin signaling plays a crucial role in the intimate relationship between lipid and glucose metabolism (Bechmann et al. 2012). In addition, the dysregulations of glucose and lipid metabolism were observed in liver diseases (Bechmann et al. 2012). Thus, we investigated the expressions of genes related to lipid and insulin metabolism in the liver. Lipoprotein lipase (LPL) (C105: 0.48 ± 0.13), PPARδ (C105: 0.78 ± 0.05), hepatocyte nuclear factor-4 α (HNF4α) (C105: 0.72 ± 0.07), and diacylglycerol acyltransferase 1 (DGAT1) (C105: 0.59 ± 0.08) gene expressions were reduced in C105 mouse livers (Figure 4A). We further investigated gene expressions of Toll-like receptor 4 (TLR4), low density lipoprotein receptor-related protein 1 (LRP-1), Akt, and MAPK that are related to inflammatory cytokine production or the clearance of Aβ (Sagare et al. 2012, Yang and Seki 2012, Wani et al. 2019, Gee et al. 2020). These gene expressions were not significantly different between C105 and WT mice (C105; TLR4: 1.03 ± 0.02, LRP1: 0.97 ± 0.13, Akt: 0.98 ± 0.09, MAPK: 1.14 ± 0.15). Furthermore, protein expression of PPARδ was reduced in contrast to those of Akt was marginally changed in C105 mouse livers (Figure 4B).

## 4. Discussion

Numerous studies have suggested glucose metabolism is associated with AD initiation or progression. In addition, obesity and metabolic impairments, such as glucose intolerance and insulin resistance, increase the risk of AD (Whitaker et al. 1997, Lloyd et al. 2010, Calsolaro and Edison 2016). Aβ burden is regionally associated with a reduction in glucose metabolism in mild cognitive impairment and early-onset AD patients (Carbonell et al. 2020). Aβ is known to induce toxicity in glucose, insulin, and lipid metabolism. Aβ deposition is linked to a reduction in glucose transporter type-1 (GLUT-1) levels in AD mouse brains (Hooijmans et al. 2007) and Aβ decreased glucose transport in cortical and hippocampal neurons by the formation of 4-hydroxynonenal, a product of lipid peroxidation (Mark et al. 1997). Aβ also impaired insulin signaling by the degradation of LRP-1, which influenced glucose metabolism and neuronal insulin signaling (Liu et al. 2015, Gali et al. 2019). In addition, Aβ induced insulin resistance by activating the JAK2/STAT3/SOCS-1 signaling pathway (Zhang et al. 2013). Furthermore, Aβ decreased lipid synthesis by reducing 3-hydroxy-3-methylglutaryl-coenzyme A reductase, which is a key enzyme for cholesterol synthesis (Grimm et al. 2007). In the present study, we found that the overexpression of carboxyl-terminal amino acid of APP in brains impaired glucose and insulin metabolism and lipid metabolism-related gene expressions. Considering food uptake was similar between WT and C105 mice, the overweight of C105 mice might be related to impaired glucose metabolism and not a change in appetite. In a previous study, C105 mice had memory impairment after 9 months (Lim et al. 2005), although they exhibited abnormal weight and glucose regulation from 9 weeks (Figure 1). We speculate that the dysregulation of glucose metabolism or being overweight is primarily observed during AD development.

Aβ penetrates the blood–brain barrier and the clearance of Aβ in the periphery promotes efflux of Aβ from the brain, thereby reducing Aβ in the brain (Roberts et al. 2014). Therefore, promoting the clearance of Aβ in peripheral tissues has been suggested to be a potential therapeutic strategy for the treatment of AD (Xiang et al. 2015). The liver is a crucial organ for reducing brain Aβ by eliminating circulating peripheral Aβ (Estrada et al. 2019). When Aβ is cleared from the brain, it is incorporated into high-density lipoprotein, transported to the liver (Sparks 2007), and then cleared by LRP-1). Thus, hepatic functions are correlated to Aβ levels, and liver cirrhosis patients with hepatitis B virus exhibited higher plasma levels of Aβ (Wang et al. 2017).

In a recent study, AD was suggested to be a liver disease of the brain (Bassendine et al. 2020), and impaired functional liver enzymes and brain glucose were suggested to be part of the AD diagnosis (Nho et al. 2019). Aβ is known to induce hepatotoxicity. It tends to accumulate in the vicinity of bile ducts and exposure to Aβ can lead to abnormal morphological and transcriptomic changes, such as biliary atresia, in human liver organoids (Babu et al. 2020). In addition, Aβ promoted the autophagy-lysosomal degradation of LRP-1 (Gali et al. 2019). Therefore, Aβ-induced hepatotoxicity may aggravate AD pathology by reducing Aβ clearance. Promoting hepatic function may attenuate AD progression or development by reducing the Aβ burden.

LPL hydrolyzes triglyceride (TG) into fatty acid and glycerol (Bechmann et al. 2012) and hepatic lipase deficiency exhibited glucose intolerance and hepatic steatosis (Andres-Blasco et al. 2015), whereas increasing LPL rescued glucose and insulin tolerance in high fat diet-induced obesity (Walton et al. 2015). PPARδ is highly expressed in hepatocytes (Hoekstra et al. 2003) and modulates glucose, fatty acid, and insulin metabolism, and insulin sensitivity (Lee et al. 2006, Cariello et al. 2021, Jang et al. 2021). HNF4α is mainly expressed in hepatocytes and plays a role in regulating glucose and lipid homeostasis as well as activating the insulin promoter (Hayhurst et al. 2001, Bartoov-Shifman et al. 2002). DGAT1 synthesizes TG and mediates lipid droplet formation (Nguyen et al. 2017, Chitraju et al. 2019), and the overexpression of DGAT1 rescued insulin resistance in diet-induced obesity (Koliwad et al. 2010). In the present study, glucose, insulin, and lipid regulatory genes were decreased in C105 mouse livers, indicating Aβ may induce overweight by the impairment of glucose and lipid metabolism in livers.

In the current study, we overexpressed a specific sequence of Aβ in mouse brains and we presented *in vivo* evidence that Aβ induced metabolic disorders and liver damage with weight gain. It would be great of interest to study the reciprocal relationship between Aβ metabolism and metabolic disorders or hepatic dysfunction, and impaired glucose metabolism during AD development in humans or AD animal models in future studies.
